# BRCAness as a prognostic indicator in patients with early breast cancer

**DOI:** 10.1038/s41598-020-78016-8

**Published:** 2020-12-03

**Authors:** Lei Liu, Yuki Matsunaga, Junji Tsurutani, Sadako Akashi-Tanaka, Hiroko Masuda, Yoshimi Ide, Rikako Hashimoto, Mayuko Inuzuka, Chie Watanabe, Kanae Taruno, Terumasa Sawada, Hiromi Okuyama, Arisa Ata, Takashi Kuwayama, Sayuka Nakayama, Yumi Tonouchi, Seigo Nakamura

**Affiliations:** 1 The Third Department of Breast Cancer, China Tianjin Breast Cancer Prevention, Treatment and Research Center, Tianjin Medical University Cancer Institute and Hospital, National Clinical Research Center of Cancer, Huanhu West Road, Hexi District, Tianjin, China; 2 Department of Breast Surgical Oncology, Showa University School of Medicine, 1-5-8 Hatanodai, Shinagawa District, Tokyo, Japan; 3 Advanced Cancer Translational Research Institute, Showa University, Tokyo, Japan

**Keywords:** Cancer, Molecular biology, Oncology

## Abstract

BRCAness is defined as a phenotypic copy of germline BRCA mutations, which describes presence of homologous recombination defects in sporadic cancers. We detected BRCAness by multiplex ligation-dependent probe amplification (MLPA) and explored whether BRCAness can be used as a predictor of prognosis. BRCAness status was classified for total 121 breast cancer patients. Forty-eight patients (39.7%) were identified as BRCAness positive. Tumors of BRCAness were more likely to be hormone receptors negative (95.8% vs. 50.7%, *P* < 0.001), nuclear grade III (76.1% vs. 48.4%, *P* = 0.001) and triple-negative breast cancer subtype (91.6% vs. 42.5%, *P* < 0.001). Five-year disease free survival (DFS) (54.0% vs. 88.0%, *P* < 0.001) and overall survival (OS) (76.3% vs. 93.1%, *P* = 0.002) were significantly lower in BRCAness patients. In neoadjuvant chemotherapy subgroup analysis, clinical response rate for taxane-based regimen was significantly lower in BRCAness patients (58.3% vs. 77.8%, *P* = 0.041). Cox regression multivariate analysis showed that BRCAness was the independent prognostic factor for DFS (HR 2.962, 95%CI 1.184–7.412, *P* = 0.020), but not for OS (HR 2.681, 95%CI 0.618–11.630, *P* = 0.188). BRCAness is associated with specific characteristics and may suggest resistance to taxane-based chemotherapy. BRCAness can be used as a negative prognostic indicator for breast cancer.

## Introduction

Germline mutations in BRCA1 and BRCA2 have been confirmed to associate with increased risk of developing breast and ovarian cancers since two decades ago^1–3^. This high risk might be related to the functions of BRCA1 and BRCA2 genes in DNA repair. In cells with germline BRCA1 or BRCA2 mutation, the DNA repair of double-strand DNA breaks (DSB) through homologous recombination (HR) was defective^4^. The exploration of BRCA1 and BRCA2 as well as homologous recombination deficient (HRD) has driven the development of targeted therapy for HRD, particularly poly (ADP-ribose) polymerase (PARP) inhibitors.

‘BRCAness’ has been reported in sporadic cancers that tumors do not have the germline mutations in BRCA but share phenotypic characteristics with tumors that carry germline BRCA mutations and consequently have defective HR^5^. Our previous research confirmed the result that same as germline BRCA mutations, BRCAness predicts resistance to taxane-containing regimens in triple negative breast cancer (TNBC) during neoadjuvant chemotherapy (NAC)^6,7^. Thus, changing regimens for BRCAness TNBC might improve their survival. At the same time, whether germline BRCA mutations can be as a prognostic indicator remains controversial^8–12^. Some studies have already shown the possibility that BRCAness is essential as a biomarker in TNBC and might be of use for predicting prognosis^13,14^.

The main purpose of our study was to further investigate whether BRCAness can be used as a prognostic factor for breast cancer with or without NAC.

## Results

### Characteristics and prognosis of all patients (n = *121)*

Of the total 121 patients, 48 (39.7%) were finally identified as BRCAness positive by MLPA. The basic characteristics stratified by BRCAness status were summarized in Table 1. No significant differences were found regarding age at diagnosis, tumor size, lymph nodal status, TNM stage or HER2 status between the two groups. However, BRCAness positive patients showed a significantly higher nuclear grade and lower hormone receptor positive rate compared to the BRCAness negative patients (*P* < 0.001 and *P* < 0.001, respectively). Thus, the proportion of TNBC subtype in BRCAness positive group was much higher than that in BRCAness negative group (91.6% vs. 42.5%, *P* < 0.001).

**Table 1:** Basic characteristics stratified by BRCAness status (n = 121).

	BRCAness (n = 48)	Non-BRCAness (n = 73)	*P* value
**Age at diagnosis**			
Range	27–74	31–75	0.268
Mean	48.0	49.1	
**Tumor size**			
T1	9 (18.8)	14 (19.2)	0.949
T2	28 (58.3)	39 (53.4)	
T3	6 (12.5)	11 (15.1)	
T4	5 (10.4)	9 (12.3)	
**Lymph node status**			
N0	27 (56.2)	34 (46.6)	0.065
N1	16 (33.3)	37 (50.7)	
N2	5 (10.4)	2 (2.7)	
**TNM stage**			
I	8 (16.7)	9 (12.3)	0.743
II	28 (58.3)	47 (64.4)	
III	12 (25.0)	17 (23.3)	
**Nuclear grade**			
I	2 (4.3)	24 (38.7)	< 0.001^a^
II	9 (19.6)	8 (12.9)	
III	35 (76.1)	30 (48.4)	
**Hormone receptor status**			
Positive	2 (4.2)	36 (49.3)	< 0.001^a^
Negative	46 (95.8)	37 (50.7)	
**HER2 status**			
Positive	2 (4.2)	10 (13.7)	0.122
Negative	46 (95.8)	63 (86.3)	
**Molecular subtype**			
Luminal	2 (4.2)	36 (49.3)	< 0.001^a^
HER2	2 (4.2)	6 (8.2)	
TNBC	44 (91.6)	31 (42.5)	

Data presented as mean and range or n (%).

^a^Statistically significant difference.

**Figure 1:**
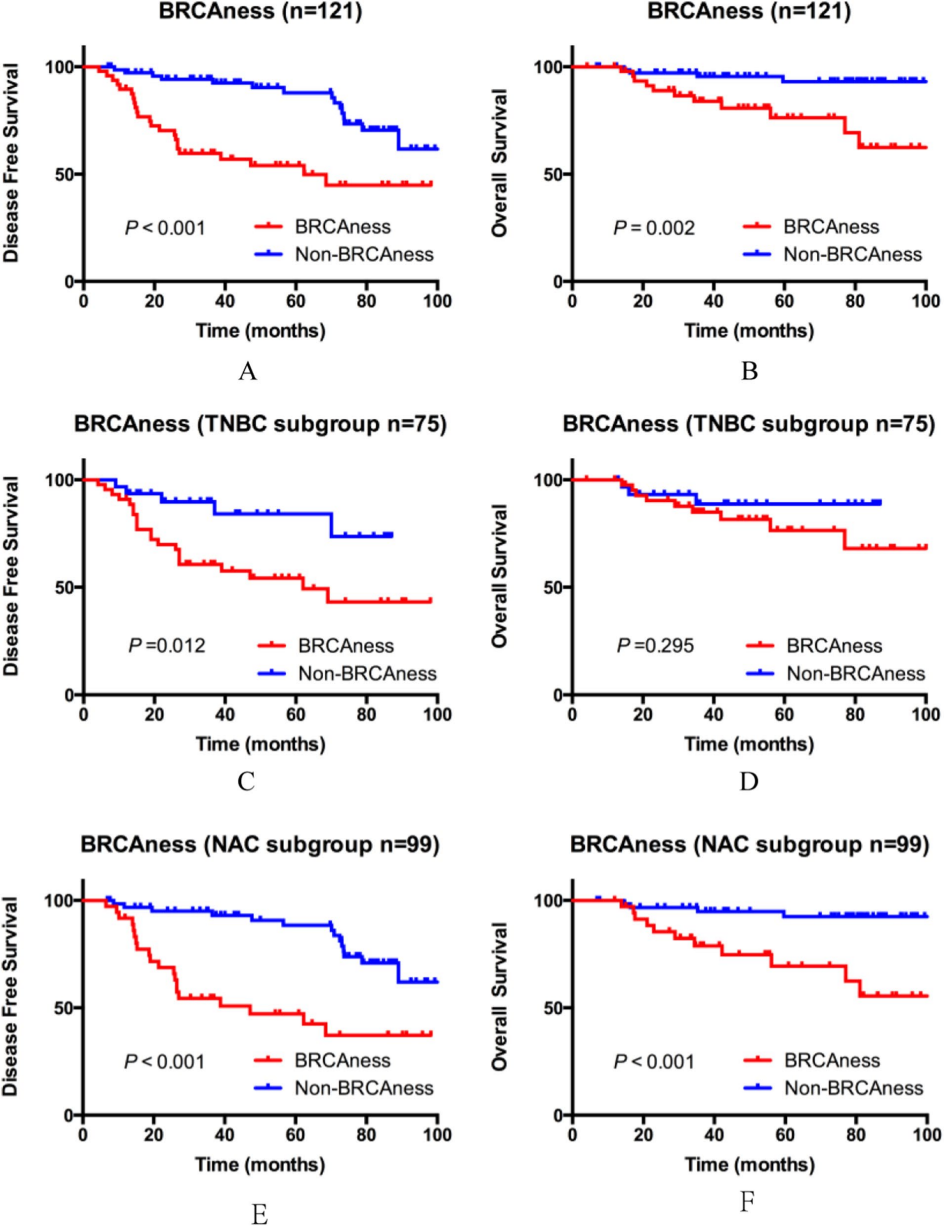
Kaplan–Meier survival curves by BRCAness.

The median follow-up of all patients was 57.7 months (range, 4.2–102.5 months). Kaplan–Meier analysis demonstrated that BRCAness positive patients showed significantly worse 5-year DFS (54.0% vs. 88.0%, *P* < 0.001) and OS (76.3% vs. 93.1%, *P* = 0.002) compared with BRCAness negative patients in whole population (Fig. 1A,B). In TNBC subgroup, BRCAness tumors also show significantly worse DFS (54.2% vs. 84.2%, *P* = 0.012) but slightly worse OS (76.5% vs. 88.8%, *P* = 0.295) (Fig. 1C,D).

**Figure 2:**
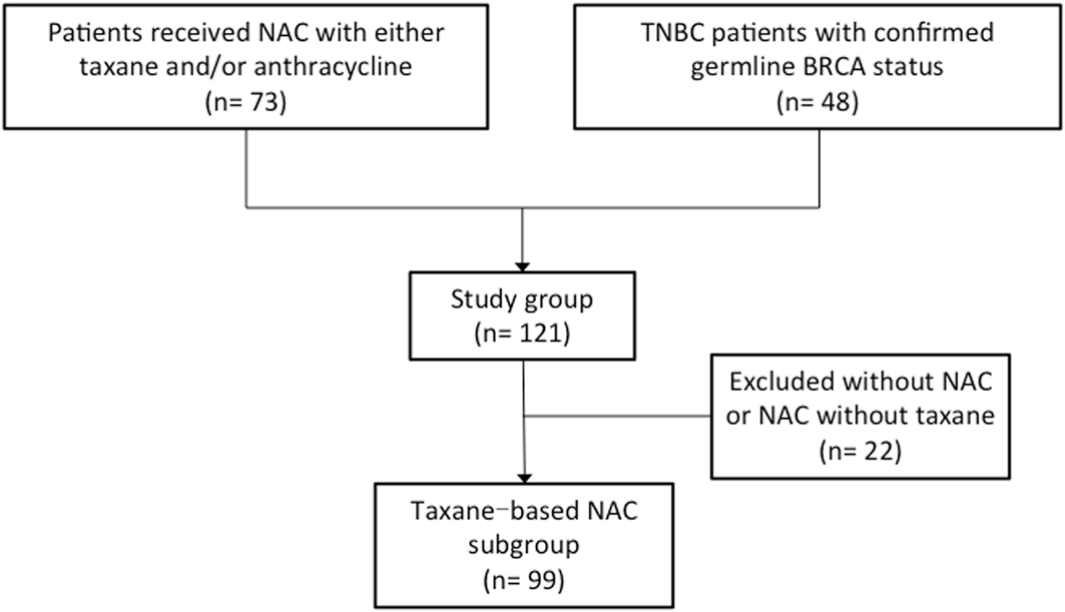
Consort diagram.

### Analysis of prognostic factors in NAC subgroup (n = *99)*

In NAC subgroup, the overall pCR rate was 17.2% (17/99), as well as cRR was 70.7% (70/99). Thirty-six patients were identified as BRCAness positive in this subgroup. As described in our previous research^7^, BRCAness was indicated significantly associated with lower cRR (58.3% vs. 77.8%, *P* = 0.041) after taxane-based regimens, but not with overall pCR rate (13.9% vs. 19.0%, *P* = 0.513).

Same as overall prognostic analysis, BRCAness positive patients still experienced significantly shorter 5-year DFS (47.2% vs. 88.4%, *P* < 0.001) and OS (69.4% vs. 92.5%, *P* < 0.001) in NAC subgroup (Fig. 1E,F). Univariate analysis of the clinicopathologic characteristics revealed that hormone receptor negative, TNBC, non-pCR and BRCAness positive were significantly associated with worse DFS, while only TNBC and BRCAness positive were associated with poorer OS (Table 2). Multivariate analysis revealed that TNBC, non-pCR and BRCAness positive were the independent and negative prognostic factor for DFS, while no factor can be as an independent factor for OS (Table 2). However, what should be noted is that because all the 15 patients who died of breast cancer were in non-pCR group, pCR could not be included as a factor in this Cox regression analysis for OS (Table 2).

**Table 2:** Cox regression analysis of DFS and OS in NAC subgroup (n = 99).

Factor	Disease free survival			Overall survival		
	HR	95% CI	*P* Value	HR	95% CI	*P* Value
**Univariate analysis**						
Tumor size (> 2 cm vs. ≤ 2 cm)	2.912	0.697–12.173	0.143	0.997	0.225–4.422	0.966
Lymph node status (positive vs. negative)	1.884	0.900–3.944	0.093	0.832	0.296–2.341	0.728
TNM stage (III vs. I–II)	1.328	0.646–2.729	0.441	0.686	0.194–2.434	0.560
Nuclear grade (III vs. I–II)	1.484	0.705–3.124	0.298	1.937	0.630–5.954	0.248
Hormone receptor (positive vs. negative)	0.272	0.121–0.610	0.002 ^a^	0.016	0.001–1.085	0.055
Molecular subtype (TNBC vs. non-TNBC)	2.676	1.304–5.492	0.007 ^a^	5.207	1.449–18.706	0.011 ^a^
Pathological response (pCR vs. non-pCR)	0.113	0.015–0.827	0.032 ^a^	N/A	N/A	N/A
Clinical response (CR + PR vs. SD + PD)	0.862	0.412–1.805	0.694	0.523	0.185–1.477	0.221
BRCAness status (positive vs. negative)	3.966	1.978–7.954	< 0.001 ^a^	6.145	1.943–19.441	0.002 ^a^
**Multivariate analysis**						
Molecular subtype (TNBC vs. non-TNBC)	2.758	1.037–7.340	0.042 ^a^	4.126	0.817–20.841	0.086
Pathological response (pCR vs. non-pCR)	0.054	0.007–0.422	0.005 ^a^	N/A	N/A	N/A
Clinical response (CR + PR vs. SD + PD)	2.034	0.910–4.547	0.083	1.215	0.411–3.597	0.724
BRCAness status (positive vs. negative)	2.962	1.184–7.412	0.020 ^a^	2.681	0.618–11.630	0.188

^a^Statistically significant difference.

In NAC subgroup, part of the patients did not have enough archival tumor tissues of CNB specimen or surgical specimen to analyze BRCAness by MLPA. Except for this part, total 42 non-pCR patients received BRCAness detection by both specimens. Twelve BRCAness patients changed to be Non-BRCAness status after NAC. These patients showed trend toward better clinical response than the other 7 patients with persistent BRCAness (75.0% vs. 28.6%, *P* = 0.074). BRCAness status remained to be stable in all the 22 Non-BRCAness patients after NAC. And this group still showed the best prognosis (Table 3). Only one Non-BRCAness patient changed to be BRCAness after NAC, so we did not include this case in Table 3. This was a TNBC patient who was resistant to taxane , and then died within 3 years.

**Table 3:** Basic characteristics and prognosis stratified by changes in BRCAness status (n = 41).

	BRCAness +/− (n = 12)	BRCAness +/+ (n = 7)	BRCAness −/− (n = 22)	*P* value 1*	*P* value 2*
**Tumor size**					
T1	1 (8.3)	1 (14.3)	2 (9.1)	0.914	0.974
T2	7 (58.3)	3 (42.9)	14 (63.6)		
T3	3 (25.0)	2 (28.6)	4 (18.2)		
T4	1 (8.3)	1 (14.3)	2 (9.1)		
**Lymph node status**					
N0	6 (50.0)	3 (42.9)	10 (45.5)	0.405	0.748
N1	6 (50.0)	3 (42.9)	11 (50.0)		
N2	0 (0)	1 (14.3)	1 (4.5)		
**TNM stage**					
I	0 (0)	1 (14.3)	1 (4.5)	0.324	0.724
II	8 (66.7)	3 (42.9)	15 (68.2)		
III	4 (33.3)	3 (42.9)	6 (27.3)		
**Nuclear grade**					
I	1 (10.0)	0 (0)	8 (44.4)	0.638	0.166
II	3 (30.0)	3 (42.9)	4 (22.2)		
III	6 (60.0)	4 (57.1)	6 (33.3)		
**Molecular subtype**					
Luminal	2 (16.7)	0 (0)	16 (72.7)	0.354	0.001^a^
HER2	1 (8.3)	0 (0)	3 (13.6)		
TNBC	9 (75.0)	7 (100.0)	3 (13.6)		
**Clinical response**					
CR + PR	9 (75.0)	2 (28.6)	18 (81.8)	0.074	0.677
PD + SD	3 (25.0)	5 (71.4)	4 (18.2)		
**Prognosis**					
5 years-DFS	40.0%	28.6%	100.0%	0.611	< 0.001^a^
5 years-OS	78.6%	83.3%	100.0%	0.757	0.001^a^

Data presented as mean and range or n (%).

*P Value 1: BRCAness +/− (n = 12) versus BRCAness +/+ (n = 7).

*P Value 2: BRCAness +/− (n = 12) versus BRCAness −/− (n = 22).

^a^Statistically significant difference.

### Correlation between BRCAness and germline BRCA mutaions (n = *63)*

Total 63 patients who were suspected of having germline BRCA mutations by physicians or genetic counsellors had received genetic test, in which 15 patients were identified as carrier of germline BRCA1 mutation, 5 patients carried germline BRCA2 mutation, and 43 were BRCA wild type. Nine patients with germline BRCA1 mutation (60.0%), 2 patients with germline BRCA2 mutation (40.0%) and 25 patients with BRCA wild type (58.1%) were identified as BRCAness positive. The difference in proportion of BRCAness was not statistically significant between patients with and without germline BRCA mutations. (55.0% vs. 58.1%, *P* = 0.815).

## Discussion

Since BRCA genes were identified, it has been confirmed that germline BRCA mutation breast cancer has some distinctive clinicopathological features compared to sporadic breast cancer. BRCA1 mutation breast cancers usually express as TNBC subtype, and associate with higher tumor grade, poorer differentiated, higher proportion of medullary and atypical medullary carcinomas^6,8,15–18^. BRCA2-mutation cancers always show a slightly increasing trend of the incidence to be lobular or tubulolobular carcinomas^19,20^.

Several preclinical and clinical studies indicated that germline BRCA mutation tumors tended to be resistant to taxane^21,22^. Similarly, correlation between BRCAness and chemotherapy efficacy have been observed in some studies. Mori and colleagues indicated that BRCAness might be a predictive factor for anthracycline-based adjuvant chemotherapy in TNBC^13^. Noguchi and colleagues and our previous research also reported that BRCAness predicted resistance to taxane-containing regimens in TNBC^6,7^. Furthermore, the current study showed that BRCAness status predicted resistance to taxane-based treatment regardless of molecular subtypes, suggesting the similar correlations of the genetic entities and sensitivity to chemotherapy in germline BRCA mutation and BRCAness. Meanwhile, similar as the characteristics of germline BRCA1 mutation, BRCAness was also associated with higher tumor grade, hormone receptor negativity, and higher proportion of TNBC subtype according to our results. Therefore, we can preliminarily conclude that there are similarities between BRCAness and germline BRCA1 mutation in clinicopathological characteristics of breast cancer.

Although according to the results of existing researches, whether germline BRCA mutations can predict the prognosis of breast cancer patients is still controversial, BRCAness showed a possibility to be a negative prognostic indicator for TNBC subtype based on the results of some previous studies^13,14^. Our result further confirmed a correlation between BRCAness and poor prognosis of all breast cancer patients regardless of molecular subtype. Furthermore, in NAC subgroup analysis, BRCAness was an independent prognostic factor for DFS. However, the reason why BRCAness could not be as an independent factor for OS might relate to the small number of involved patients and low mortality rate. Therefore, a bigger sample size is necessary for our further research to establish this trend and effect on OS.

In NAC subgroup, part of the patients changed their BRCAness status after receiving NAC. Although we highlighted this change in our result part, we still believe that it cannot be as a key point because of the small number of patients. In the analysis of relevance between the change and characteristics, BRCAness patients who changed to be Non-BRCAness status after NAC showed better clinical response than the persistent BRCAness patients. We considered it might be because of the reason that more normal-cell contamination after NAC affected BRCAness results. But this change might also suggest that HR function could be restored during NAC, thus the sensitivity of taxane improved in this subgroup. However, the prognosis was more related to initial BRCAness status.

In our research preparation period, we anticipated a higher percentage of BRCAness positive in germline BRCA mutation breast cancer compared to sporadic breast cancer. However, our final result confirmed that the difference in proportion of BRCAness was not statistically significant between patients with and without germline BRCA mutations. It might suggest that germline BRCA mutations and BRCAness detected by somatic cell were quite different. As described in the conclusion of TNT trail^23^, defects in HR might be revertible, while mutational signatures as a permanent ‘scar’ of prior would not be expected to disappear. BRCA genes and HRD were now known as the principle of PARP inhibitor targeted therapy^4^. Although our research did not cover this part, we still believe that PARP inhibitors may also be beneficial for BRCAness patients, which requires new clinical trials to further confirm.

BRCAness is associated with specific characteristics of breast cancer and may suggest resistance to taxane-based chemotherapy. BRCAness can also be used as a negative prognostic indicator for all breast cancer patients regardless of molecular subtype.

## Patients and methods

### Patients

This study was approved by the ethical committee of Showa University. All methods were carried out in accordance with relevant guidelines and regulations. Informed consent was obtained from all subjects. One hundred and twenty one patients who were diagnosed of early invasive breast cancer from July 2005 to July 2017 at Showa University Hospital Breast Center were involved in this study. All the patients were at TNM stage I-III, which we defined it as early breast cancer due to the opportunity for surgery. Patients were involved from two different parts. Seventy-three patients were enrolled according to the principle that all the patients had received NAC with taxane and/or anthracycline for primary breast cancer from October 2010 to March 2013 at Showa University Hospital Breast Center. Most of these patients had been involved in a randomized controlled trial in which we compared the efficacy of docetaxel and albumin-bound paclitaxel as NAC. The other 48 patients were randomly selected from TNBC patients with confirmed germline BRCA mutation status. Excluding 22 patients without NAC or NAC without taxane, a total of 99 patients had received taxane-based NAC (Fig. 2).

Ultrasonography and magnetic resonance imaging (MRI) were performed before treatment, and at the end of first and second cycle of NAC. For patients whose tumor was determined to progress after two cycles of NAC, tanaxe-based regimen would be changed to a second-line regimen, or surgery would be performed immediately. Clinical response for taxane was based on imaging before NAC and after the last cycle of taxane-based NAC, using the Response Evaluation Criteria in Solid Tumors^24^. Clinical response rate (cRR) was summarized the rate of clinical complete response (cCR) and clinical partial response (cPR).

Survival endpoints of this study were disease-free survival (DFS) and overall survival (OS). DFS was defined as the interval from first treatment to recurrence or metastasis. OS was defined as the interval from first treatment to death of any cause.

### Pathology

In this study, the pathological reports of the routine surgical specimens were used. Tumor, node, and metastasis staging of breast cancer was performed following the Cancer Staging Manual (8th edition) of the American Joint Committee on Cancer (2016). Tumors were graded using the modified Black’s nuclear grading system.

Molecular classification was based on the expression of estrogen receptor (ER), progesterone receptor (PR), Ki-67, and human epidermal growth factor receptor 2 (HER2). Expression of ER, PR, HER2 and Ki-67 was first examined by immunohistochemistry and scored by pathologists. Sections with ≥ 1% of cells expressing ER or PR were scored as positive. The tumors were considered HER2-positive if immunohistochemical analysis score was greater than 3 or amplification of HER2 gene was confirmed by fluorescence in situ hybridization (FISH), as the American Society of Clinical Oncology guidelines. Ki-67 staining was scored as the percentage of cells with positive nuclear signals (0–100%). Sections with > 20% of positive cells were defined as having a high level of Ki-67 expression. Molecular subtypes were defined following St. Gallen Breast Classification 2017.

In determination of pathological efficacy, pathological complete response (pCR) was defined as complete remission of the invasive components of cancer in the breast and lymph nodes^25^.

### MLPA method

BRCAness was detected by examination of formalin-fixed, paraffin-embedded (FFPE) core needle biopsy (CNB) specimens and/or surgical specimens. DNA was isolated from the tumor using the QIAamp DNA FFPE Tissue Kit (Qiagen, Hilden, Germany) after macro dissection. BRCAness was classified by multiplex ligation-dependent probe amplification (MLPA) with the Probemix P376-BRCA1ness (MRC-Holland, Amsterdam, The Netherlands)^26^. MLPA was performed at Falco Biosystems (Kyoto, Japan) according to the manufacturer’s instructions. BRCAness assays for the first 73 patients were performed at no cost under a collaborative study contract. The relative copy number ratios for the 38 target-specific probes, compared with the reference samples of human genomic DNA (Promega, Madison, WI), were calculated by Coffalyser.Net software and were used for the prediction analysis for microarrays, with the training set generated by MRC-Holland. Laboratory scientists who analyzed BRCAness status were unaware of the patients’ clinical information. The cutoff ratio for BRCAness positivity in this study was 0.5. For patients who had both BRCAness results of CNB specimen and surgical specimen, if one of the results was positive, the patient was defined as BRCAness positive.

### Germline BRCA analysis

Sixty-three patients who were suspected of having germline BRCA mutations by physicians or genetic counsellors had received genetic detection according to National Comprehensive Cancer Network guidelines. Detection was performed at Falco Biosystems (Kyoto, Japan) using direct sequencing method on blood samples. Positivity of germline BRCA mutation was defined as presence of pathogenic mutations and/or possible pathogenic mutations.

### Statistical analysis

Data was processed using a Statistical Package for the Social Sciences (version 20.0). Survival curves were plotted using GraphPad Prism (version 6.0). Categorical variables were compared using a Chi-square test. Age difference between groups was analyzed by a non-parametric test. 5-year DFS and OS were generated using the Kaplan–Meier method and were compared using the log-rank test. Hazard ratios were calculated using Cox proportional hazards regression. *P*-value lower than 0.05 was considered significant.

## Data Availability

The datasets during and/or analysed during the current study are available from the corresponding author on reasonable request.

## Abbreviations

MLPA: Multiplex ligation-dependent probe amplification
DFS: Disease free survival
OS: Overall survival
DSB: Double-strand DNA breaks
HR: Homologous recombination
HRD: Homologous recombination deficient
PARP: Particularly poly (ADP-ribose) polymerase
TNBC: Triple negative breast cancer
NAC: Neoadjuvant chemotherapy
MRI: Magnetic resonance imaging
cRR: Clinical response rate
cCR: Clinical complete response
cPR: Clinical partial response
ER: Estrogen receptor
PR: Progesterone receptor
HER2: Human epidermal growth factor receptor 2
FISH: Fluorescence in situ hybridization
pCR: Pathological complete response
FFPE: Formalin-fixed, paraffin-embedded
CNB: Core needle biopsy
